# THE AGING RESEARCH IN CRIMINAL JUSTICE HEALTH (ARCH) NETWORK: WHERE WE’VE BEEN AND WHERE WE ARE GOING

**DOI:** 10.1093/geroni/igae098.1544

**Published:** 2024-12-31

**Authors:** Lisa Barry, Jennifer James, Melissa Gerald, Fernando Murillo

**Affiliations:** Chair; Co-Chair; Discussant; Discussant

## Abstract

Established in 2019, the NIA-funded “Aging Research in Criminal Justice and Health (ARCH) Network” brings together a multi-disciplinary, international group of researchers and community leaders dedicated to evaluating the life course experiences of justice-involved older adults and translating that research into policy and practice. In this symposium, we will celebrate the ARCH Network’s accomplishments over the past five years by highlighting inspirational investigators, community leaders, and pilot projects that have helped to advance science in this emerging research area. The first presentation will provide a brief overview of the ARCH Network and its mission, highlighting the journey from development to inception to continual growth. The second presentation will focus on the impact of the ARCH Network on the career trajectory of early-stage investigators. From the perspective of a doctoral student, we will learn about how the ARCH network is a pipeline model for advancing the careers of scholars interested in health equity and policy research focused on justice-involved older adults. The third presentation will highlight the practice interventions and community resources that have emerged from health-equity focused, collaborative, aging research on incarceration. The fourth presentation will discuss policy-related impacts and solutions for justice-involved older adults. The final presentation will focus on the impact of the ARCH Network from the perspective of an individual with lived experience and will highlight the vision for the ARCH Network moving forward. Following the presentations, we look forward to an interactive discussion led by the NIA Program Officer for the ARCH Network. Incarceration and Aging Interest Group Sponsored Symposium

